# Clinical research of the value of high-risk CTV setting on intensity-modulated radiotherapy for stage IIB-IVA cervical cancer

**DOI:** 10.1186/s12885-023-10931-1

**Published:** 2023-05-27

**Authors:** Yuqi Jiang, Jing Wang, Peng Jiang, Xiang Wang, Lei Zhang, Yongchun Zhang

**Affiliations:** Department of Radiation Oncology, The Affiliated Hospital of Qingdao University, 16 Jiangsu Road, Qingdao, 266003 China

**Keywords:** Uterine cervical neoplasms, Neoplasm recurrence, Intensity modulated radiotherapy, Adverse effects, Simultaneous integrated boost intensity-modulated radiotherapy, High-risk CTV

## Abstract

**Background:**

This study aims to evaluate the clinical efficacy and side effects of setting up a high-risk clinical target volume (CTV-hr) alongside simultaneous integrated boost intensity-modulated radiotherapy (IMRT-SIB) in patients diagnosed with stage IIB-IVA cervical cancer.

**Methods:**

This study retrospectively analysed patients with stage IIB-IVA cervical cancer who received radical radiotherapy at the Affiliated Hospital of Qingdao University between November 2014 and September 2019. The patients were divided into experimental and control groups based on whether CTV-hr was set. All patients received a combined treatment of radiotherapy and chemotherapy. The dosage for paclitaxel was 135 mg/m^2^, while for cisplatin it was 75 mg/m^2^ or for carboplatin it was AUC 4–6, given in a cycle of 21 days. Radiotherapy (RT) included external beam radiation therapy (EBRT) and intracavitary brachytherapy (ICBT). In the control group, positive lymph nodes (GTV-n) were treated at a dose of 58–62 Gy/26–28 fractions(f), while clinical target volumes (CTV) were treated with a dose of 46–48 Gy/26-28f. The experimental group received a simultaneous integrated boost (SIB) to CTV-hr at a dose of 54–56 Gy/26-28f, with the same CTV and GTV-n as the control group. Both groups were combined with brachytherapy with a total dose (EQD2, the equivalent dose in 2 Gy/f) of 80-90 Gy. The study measured objective remission rate (ORR), 3-year progression-free survival (PFS) rate, 3-year overall survival (OS) rate, recurrence rate, and side effects as endpoints.

**Results:**

The study enrolled 217 patients, with 119 in the experimental group and 98 in the control group. Results showed that the experimental group had a higher 3-year OS rate (87.4% vs. 71.4%, *p* = 0.001) and 3-year PFS rate (72.3% vs. 51.0%, *p* = 0.000) compared to the control group. Additionally, the experimental group had significantly lower rates of overall recurrence (26.1% vs. 50.0%, *p* = 0.003), in-field recurrence (15.1% vs. 36.7%, *p* = 0.000), and out-field recurrence(13.4% vs. 35.7%, *p* = 0.000) compared to the control group. All observed differences were found to be statistically significant. However, the experimental and control groups had no statistically significant difference in ORR and radiological side effects, such as radiation cystitis and enteritis (*p* > 0.05).

**Conclusions:**

Setting CTV-hr and performing IMRT-SIB on patients with stage IIB-IVA cervical cancer effectively increased the 3-year OS rate, 3-year PFS rate and reduced recurrence rate, with no significant differences in side effects.

## Background

Cervical cancer is a prevalent malignant tumor in China's female reproductive system, which poses a significant threat to women's health and life [1]. Radical chemoradiotherapy is the preferred treatment option for locally advanced cervical cancer [2]. However, despite this treatment, some patients still experience recurrence leading to death. The 3-year PFS rate is 74.4% (95% CI, 68.0%-79.8%) [3], and the 5-year overall survival rate is approximately 70% [2]. The sites of local recurrence were the central pelvis (36.7%), vaginal vault (20.5%), pelvic wall (9.5%), and lymph nodes (4.9%) [4]. Therefore, improving the local control rate of cervical cancer is a current focus of clinical research and treatment. In recent years, IMRT-SIB has emerged as a promising technique for delivering a higher radiation dose to the target volume and reducing the irradiated volume and dose to the surrounding normal tissues without extending the external irradiation time [5, 6]. This leads to a reduction in side effects and better therapeutic outcomes. However, there is currently no research on SIB to areas with high recurrence rates. Current clinical studies focus on SIB to the positive lymph nodes. Because of this background, we retrospectively analyzed 217 patients with locally advanced cervical cancer who underwent radical chemoradiotherapy at the Radiotherapy Department of Qingdao University Hospital in this study. We compared the clinical efficacy and side effects of the two groups of patients, and the results are reported as follows.

## Methods

The study cohort included patients with stage IIB-IVA cervical squamous cell carcinoma or adenocarcinoma who underwent radical chemoradiotherapy at the Affiliated Hospital of Qingdao University from November 2014 to September 2019. The patients were divided into two groups: the experimental group (CTV-hr was set in the radiotherapy target volume) and the control group (CTV-hr was not set).By the FIGO 2018 principles of cervical cancer staging, all patients were restaged. Any lymph node metastasis(LNM) cases were classified as stage IIIC. If only pelvic lymph nodes were positive, they were classified as stage IIIC1; if para-aortic lymph nodes(PALN) were involved, they were classified as stage IIIC2. Before treatment, all patients underwent routine blood, liver and kidney function, electrolyte, and other relevant laboratory and imaging tests. The study excluded individuals with serious underlying diseases, as well as those with other tumors and significant abnormalities in heart, lung, liver, and kidney function. None of the patients had contraindications to radiotherapy and chemotherapy treatment, and all signed an informed consent form. The ethics committee approved the study.

### Radiotherapy

RT included EBRT and ICBT in both groups. EBRT used IMRT with 6 mV X-rays. Before ERBT, CT and contrast-enhanced CT scans were required for simulation localization. The scanning layer should be 5 mm thick. The upper border should be set at the upper edge of the 10th thoracic vertebra, while the lower border should be set at the lower 10 cm of the ischial tuberosity. The patients emptied the bladder 2 h before the radiotherapy simulation localization and filled the bladder with 500–800 mL of water and 20 mL of pantothenic glucosamine 30–40 min before the radiotherapy simulation localization to reduce the volume of the small intestine. They were placed in the prone position and secured with thermoplastic film. According to the principle of RTOG [7], the target volume and organs at risk (OARs) of the experimental and control groups were outlined respectively.

The definition and dose of ERBT target volume in the control group are as follows .GTV-n is defined as lymph nodes with a diameter ≥ 5 mm on CT/MRI or lymph nodes more than 3 in the same plane with blurred edges and liquefied centers on CT/MRI, or lymph nodes with abnormal metabolic activity on PET/CT or PET/MR. CTV definition: the uterine body, the cervix, side of the uterus, part or all of the vagina and the partial lymphatic drainage areas. If the common iliac lymph nodes or PALN metastasis has occurred, extended field (para-aortic field) radiotherapy (EFRT) is required. The upper boundary of the radiation field is at the level of the left renal vein or the upper margin of the first lumbar vertebrae. The lower boundary is connected with the pelvic radiation field. The left boundary is the left 2 cm of the abdominal aorta, the left psoas major muscle. The right boundary is the right margin of the inferior vena cava. The anterior boundary is the anterior 7 mm of the blood vessel while avoiding the small intestine as much as possible. The posterior boundary is the anterior edge of the vertebral body. If there are any visibly enlarged lymph nodes on imaging, the extended field must include the area of the enlarged lymph nodes, which should be expanded by 5 mm. PTV definition: CTV extended 5 mm forming the planned target volume. Dose: 46–48 Gy/26–28 fraction (f) was given to the CTV area and 58–62 Gy/26–28f was given to GTV-n.

The definition and dose of ICBT target volume in the control group are as follows. High-risk clinical target volume for tumors (CTV-T_HR_): the areas with the highest risk of recurrence identified by physical examination and imaging examination, including residual tumors, all cervix and diseased tissues after ERBT. The term 'diseased tissue' refers to the gray area around the tumor on MRI images after ERBT, including the edema and fibrosis of the original tumor after ERBT, the residual mass touched by physical examination, and the changes of residual mucosa visible to the naked eye. Intermediate-risk clinical target volume(CTV-T_IR_): the primary tumor lesion before ERBT and the external extension of CTV-T_HR_ (The upper, lower, left and right sides were enlarged by 10 mm but the anterior and posterior sides were 5 mm). Dose: the lowest dose received by 90% of the target volume (D90) of CTV-T_HR_ is 28-30 Gy/5f. D90 of CTV-T_IR_ is 18-20 Gy/5f.

The definition and dose of ERBT target volume in the experimental group are as follows. CTV-hr: the uterine body, the cervix, side of the uterus, part or all of the vagina and positive lymph nodes drainage area. GTV-n, CTV and PTV are defined as the same as the control group. Dose: the CTV area received an external dose of 46–48 Gy/26–28 f, the GTV-n received an external dose of 58–62 Gy/26-28f and the CTV-hr with SIB was 54–56 Gy/26–28 f.

The definition and dose of ICBT target volume in the experimental group are as follows. CTV-T_HR_, CTV-T_IR_ are defined as the same as the control group. Dose: 22-24 Gy/4f for CTV-T_HR_ D90 and 14-16 Gy/4f for CTV-T_IR_ D90.

**Fig. 1:**
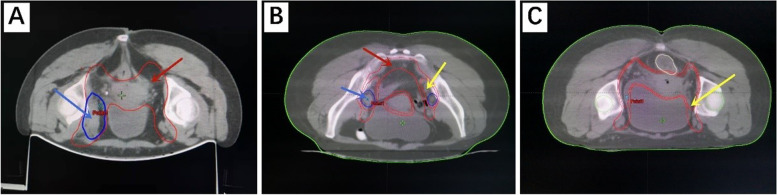
**A**. the control group target volume; **B**-**C**. the experimental group target volume. Red arrow: CTV. Yellow arrows: CTV-hr. Blue arrows: positive lymph nodes

ERBT is given five times a week and guided by IGRT. When the primary tumor has reduced to less than 3 cm after 15 fractions or when ERBT is completed, 3D ICBT is utilized in combination. The EQD2 of ERBT combined with ICBT is 80–90 Gy, reaching 85–90 Gy for patients with a tumour diameter > 4 cm or poor tumour regression during treatment. Figure 1 shows the target volume for ERBT.

### Chemotherapy

Both groups of patients received treatment with a combination of paclitaxel and either cisplatin or carboplatin. The treatment regimen consisted of paclitaxel at a dose of 135 mg/m^2^, combined with either cisplatin at a dose of 75 mg/ m^2^ or carboplatin at an AUC of 4 to 6, administered every 21 days.

### Post-treatment follow-up

Patient follow-up was conducted regularly after treatment until October 2022. Survival and progression-free dates are calculated from the date of diagnosis. The follow-up schedule included reviews every 2 months for the first year, every 3 months from year one to year two, and every six months thereafter. At each follow-up visit, patients underwent ultrasound, CT, MRI, and tumour marker tests. Disease progression was determined using the Response Evaluation Criteria in Solid Tumors (RECIST, version 1.1) and side effects were assessed according to the Common Terminology Criteria for Adverse Events version 4.0 (CTCAE version4.0). In medical terms, recurrent disease refers to the emergence of a new or progressive lesion at any site, which is identified by pathological or radiological methods during follow-up. When this recurrence happens within the radiation treatment field, it is known as an in-field recurrence. Conversely, if it occurs outside the radiation treatment field, it is called an out-field recurrence. The endpoints for assessing the effectiveness of treatment include ORR, 3-year PFS rate, 3-year OS rate, recurrence rate, and incidence of side effects.

### Statistical analysis

The data was analyzed using SPSS 26.0 statistical software. Either the χ2 test or the t-test was used for analysis. The Kaplan–Meier method was used to plot survival curves, which were then compared using the log-rank test. A *p*-value of less than 0.05 was considered statistically significant.

## Results

The study included 217 patients, with 119 in the experimental group and 98 in the control group. The median follow-up time for the entire cohort was 57 months (range: 8–95 months), with the experimental group having a median follow-up time of 56 months (range: 8–95 months) and the control group having a median follow-up time of 58 months (range: 10–95 months) (*p* = 0.546). The general clinical data of both groups were comparable and not statistically different (Refer to Table 1 for further details). The average number of chemotherapy cycles in the experimental group was 3.51 ± 1.16, while in the control group it was 3.73 ± 1.27, with no statistically significant difference between the two groups (*p* = 0.18). Similarly, the total radiation treatment dose received by the experimental group was 83.41 ± 2.46 Gy and by the control group was 83.03 ± 2.13 Gy, with no significant difference between the two groups (*p* = 0.229).

**Table 1:** Patient characteristics

Characteristic	EG(*n* = 119)	CG(*n* = 98)	*p* -value
Histology			0.840
Squamous	109	89	
Adenocarcinoma	10	9	
Median age, y (range)	54(35–78)	54(32–78)	0.428
Chemotherapy			0.394
Paclitaxel + Cisplatin	108	92	
Paclitaxel + Carboplatin	11	6	
FIGO stage			0.229
IIb	36	20	
IIIb	13	14	
IIIc1	54	42	
IIIc2	9	15	
IVa	7	7	
Maximum tumor diameter (cm)	4.42 ± 0.94	4.51 ± 1.56	0.636
Pretreatment hemoglobin (g/L)	114.59 ± 17.10	112.87 ± 16.34	0.452

*EG* The experimental group, *CG* The control group

### Short-term effects

The experimental group had a higher complete response (CR) rate (81.5% vs 70.4%; *p* = 0.055) and ORR(91.6% vs 90.8%; *p* = 0.484). However, the experimental group had a lower partial response (PR) rate (10.6% vs 18.4%; *p* = 0.079) and stable disease (SD) rate (8.4% vs 11.2%; *p* = 0.484). Despite these differences, the statistical significance was not established (Table 2).

**Table 2:** Short-term efficacy

	EG( *N*= 119),n(%)	CG(*N* = 98),n(%)	*p* -value
CR	97 (81.5%)	69 (70.4%)	0.055
PR	12 (10.6%)	18 (18.4%)	0.079
SD	10 (8.4%)	11 (11.2%)	0.484
PD	0(0%)	0(0%)	
ORR	109 (91.6%)	88 (90.8%)	0.484

*EG* The experimental group, *CG* The control group, *ORR* Overall response rate, CR + PR, *CR* Complete response, *PR* Partial response, *SD* stable disease, *PD* progressive disease

### Treatment outcome

The experimental group showed a significant reduction in overall recurrence rate (26.1% vs 50%;*p* = 0.003), in-field recurrence rate (15.1% vs 36.7%; *p* = 0.000) and out-field recurrence rate (13.4% vs 35.7%; *p* = 0.000) compared to the control group. The results are presented in Table 3.

**Table 3:** Treatment outcome

	EG(*N* = 119),n(%)	CG(*N* = 98), n(%)	*p* -value
overall recurrence	31(26.1%)	49 (50.0%)	0.003
In-field recurrence	18 (15.1%)	36 (36.7%)	0.000
Out-field recurrence	16 (13.4%)	35 (35.7%)	0.000

*EG* The experimental group, *CG* The control group

In this study, we investigated the effectiveness of IMRT with SIB to CTV-hr in treating different stages of cervical cancer. Our findings showed that in stage IIB and IIIB, the experimental group had significantly lower recurrence rates than the control group. Specifically, the experimental group had a lower overall recurrence rate (26.5% vs. 50%, *p* = 0.029) and an in-field recurrence rate(12.2% vs. 35.3%, *p* = 0.012) and an out-field recurrence rate(18.4% vs. 47.1%, *p* = 0.005). In stage IIIC1, the experimental group had a lower overall recurrence rate (20.4% vs 42.9%, *p* = 0.017), in-field recurrence rate (13.0% vs 33.3%, *p* = 0.017) and out-field recurrence rate (7.4 vs 21.4%, *p* = 0.046) than the control group, all with statistically significant differences. In stage IIIC2 and IVA, the *p-*values were greater than 0.05, indicating that none of the three variables were statistically significant. This information can be found in Table 4.

**Table 4:** Treatment outcome after the grouping

	EG,n/N(%)	CG,n/N(%)	*p* -value
Stage IIB and IIIB			
Overall recurrence	13/49 (26.5%)	17/34 (50%)	0.029
In-field recurrence	6/49 (12.2%)	12/34 (35.3%)	0.012
Out-field recurrence	9/49 (18.4%)	16/34 (47.1%)	0.005
Stage IIIC1			
Total recurrence	11/54 (20.4%)	18/42 (42.9%)	0.017
In-field recurrence	7/54 (13.0%)	14/42 (33.3%)	0.017
Out-field recurrence	4/54 (7.4%)	9/42 (21.4%)	0.046
Stage IIIC2			
Total recurrence	5/9 (55.6%)	10/15 (66.7%)	0.687
In-field recurrence	4/9 (44.4%)	8/15 (53.3%)	1
Out-field recurrence	2/9 (22.2%)	7/15 (46.7%)	0.389
Stage IVA			
Total recurrence	2/7(28.6%)	4/7 (57.1%)	0.592
In-field recurrence	1/7 (14.3%)	2/7 (28.6%)	1
Out-field recurrence	1/7(14.3%)	3/7(42.9%)	0.559

*EG* The experimental group, *CG* The control group

### Overall survival and progression-free survival

**Fig. 2:**
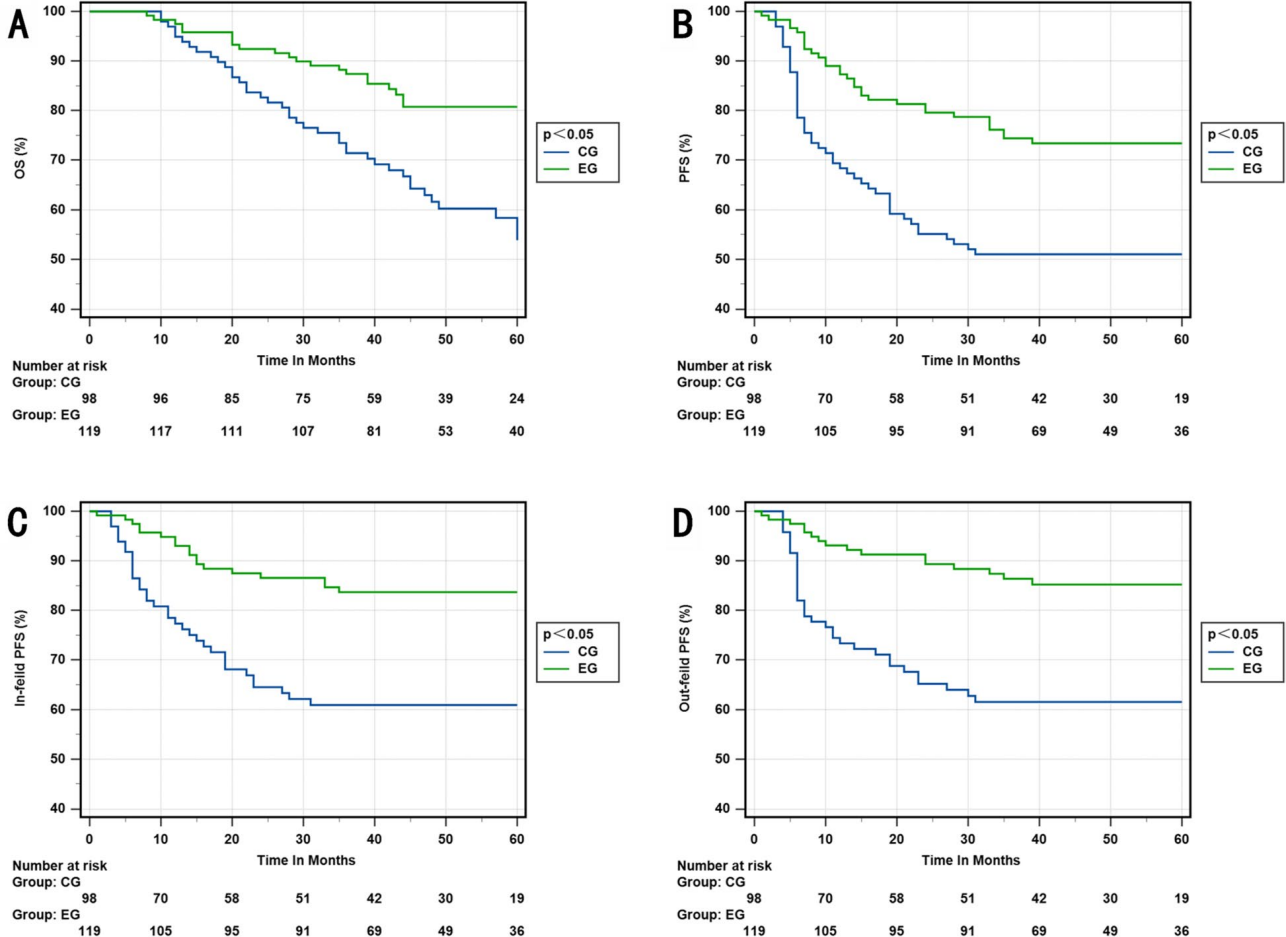
**A **OS in the EG and CG. **B** PFS rate in the EG and CG. **C** In-field PFS rate in the EG and CG. **D** Out-field PFS rate in the EG and CG. EG, experimental group; CG, control group

Figure 2 displays the KM survival curves for the two different regimens. The results show a significant improvement in OS (3-year OS rate, 87.4% vs. 71.4%; *p* = 0.001) and PFS (3-year PFS rate, 72.3% vs. 51.0%; *p* = 0.000), as well as in-field PFS (3-year in-field PFS rate, 83.7% and 60.9%; *p* = 0.000) and out-field PFS (3-year out-field PFS OS rate, 86.4% and 61.5%; *p* = 0.000).

**Fig. 3:**
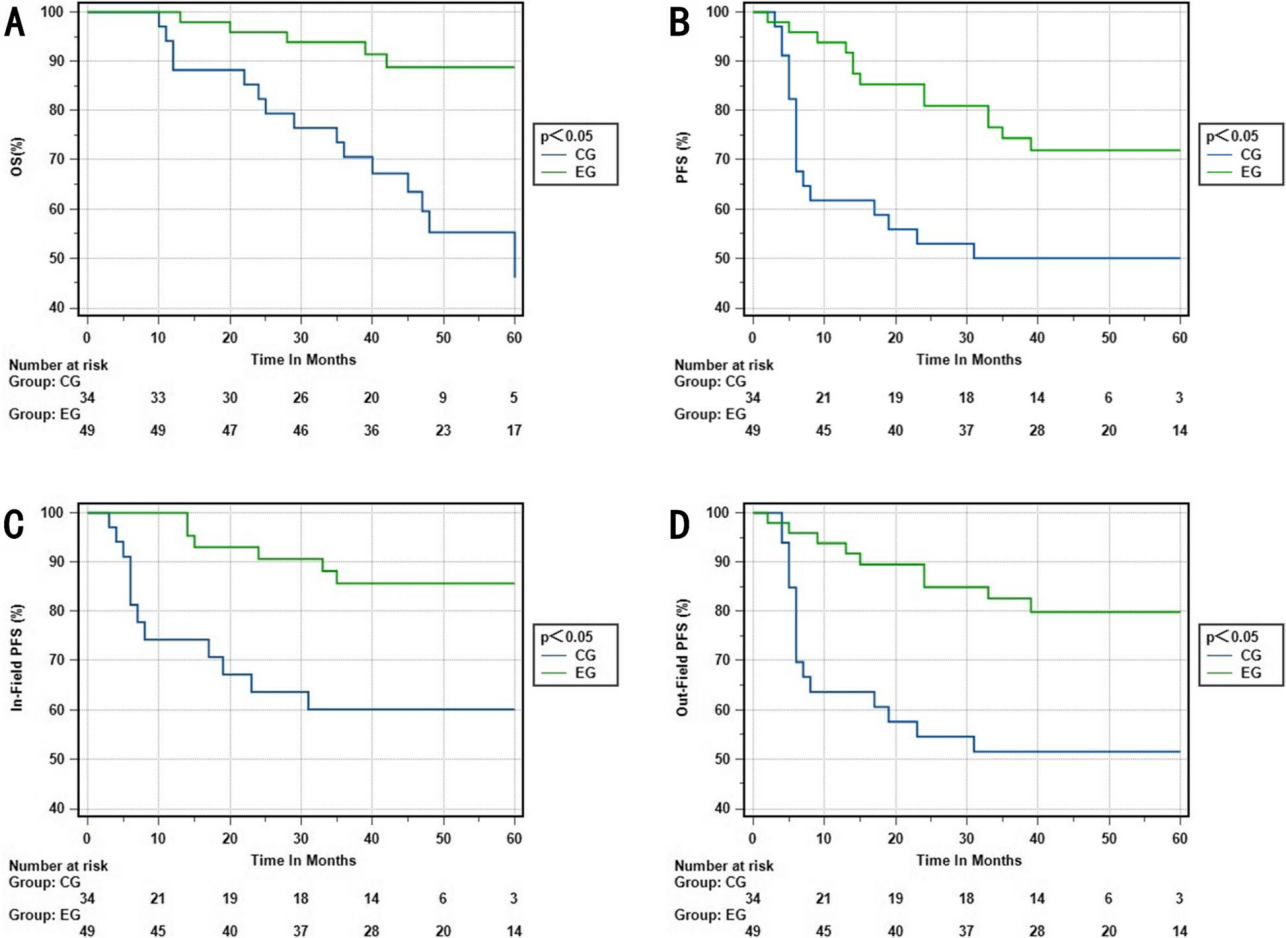
**A** OS rate of stage IIB and IIIB in the EG and CG. **B** PFS rate of stage IIB and IIIB in the EG and CG. **C** In-field PFS rate of stage IIB and IIIB in the EG and CG. **D** Out-field PFS of stage IIB and IIIB in the EG and CG. EG, experimental group; CG, control group

**Fig. 4:**
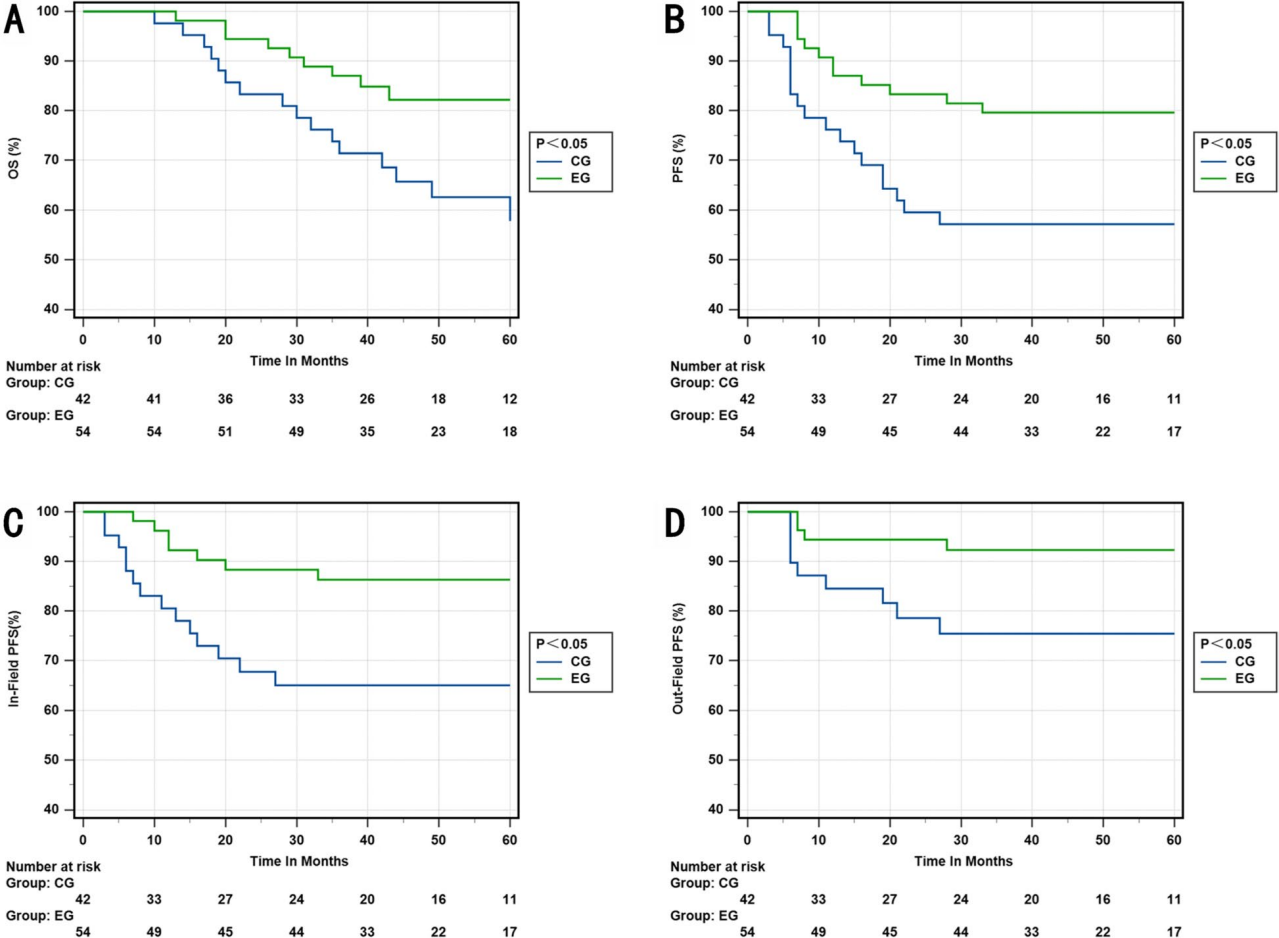
**A** OS rate of stage IIIC1 in the EG and CG. **B** PFS rate of stageIIIC1 in the EG and CG. **C** In-field PFS rate of stage IIIC1 in the EG and CG. **D** Out-field PFS of stage IIIC1 in the EG and CG. EG, experimental group; CG, control group

**Fig. 5:**
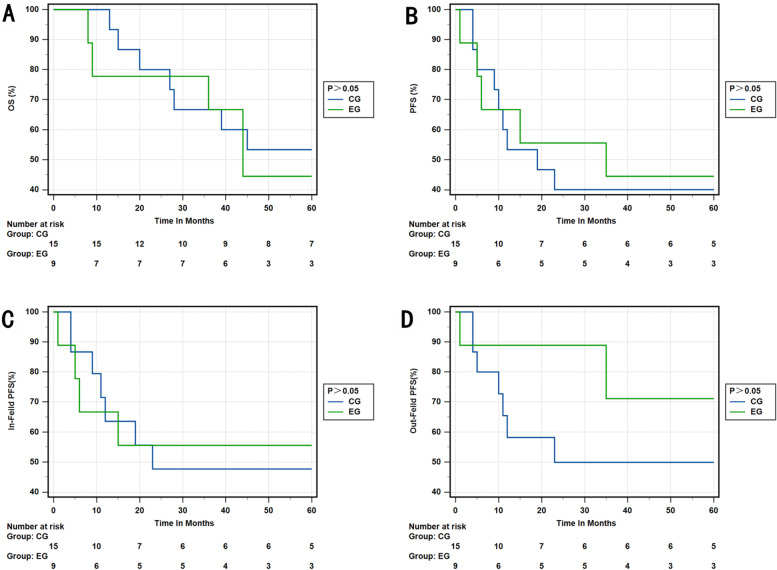
**A** OS rate of stage IIIC2 in the EG and CG. **B** PFS rate of stage IIIC2 in the EG and CG. **C** In-field PFS rate of stage IIIC2 in the EG and CG. **D** Out-field PFS of stage IIIC2 in the EG and CG. EG, experimental group; CG, control group

**Fig. 6:**
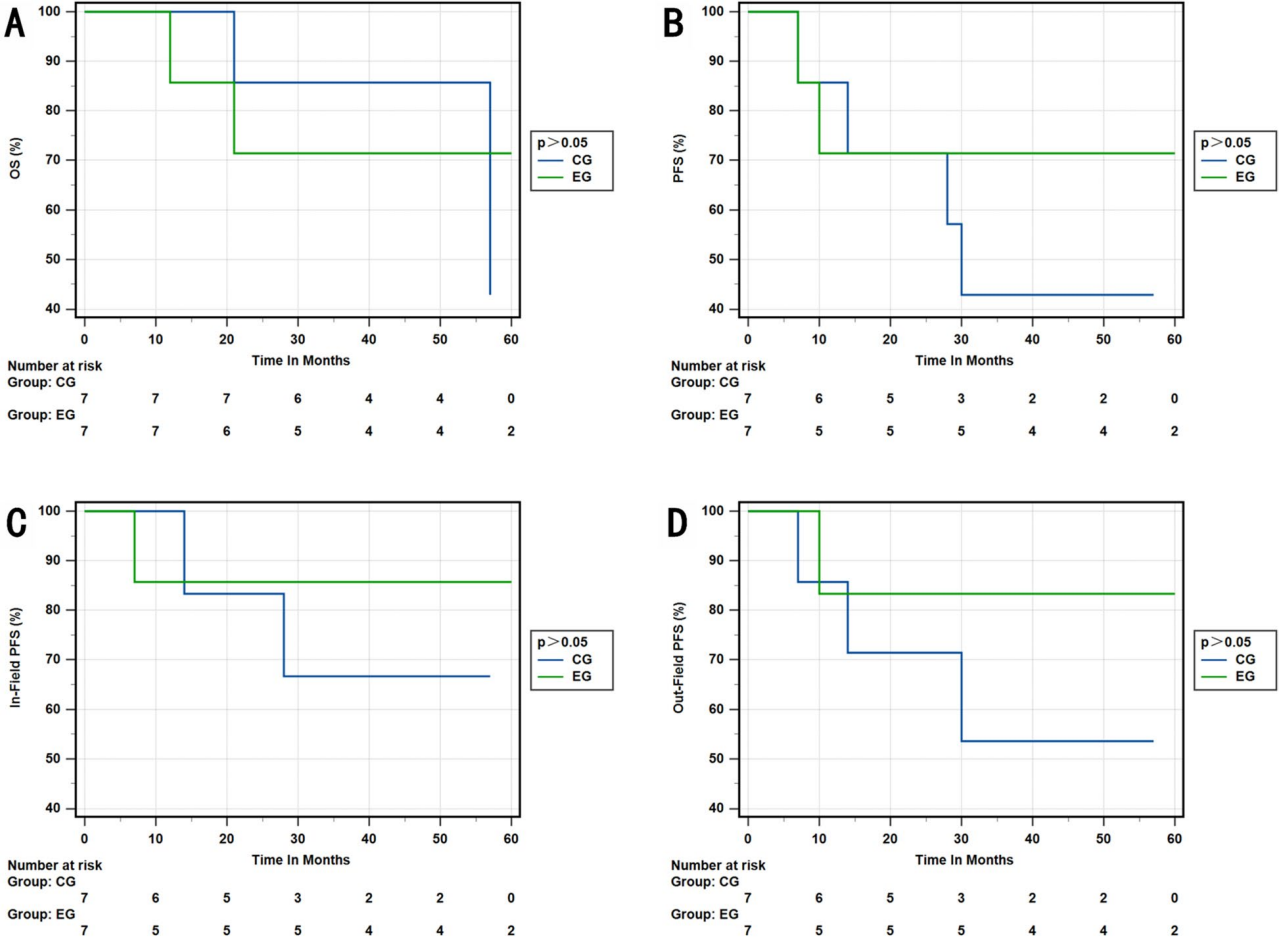
**A** OS rate of stage IVA in the EG and CG. **B** PFS rate of stage IVA in the EG and CG. **C** In-field PFS rate of stage IVA in the EG and CG. **D** Out-field PFS of stage IVA in the EG and CG. EG, experimental group; CG, control group

We conducted a detailed analysis on the impact of setting CTV-hr with IMRT-SIB on different stages of cervical cancer. Our findings revealed that the experimental group outperformed the control group in terms of 3-year OS rate, 3-year PFS rate, 3-year in-field PFS rate, and 3-year out-field PFS rate in stage IIB and IIIB (*p* = 0.001; *p* = 0.014; *p* = 0.005; *p* = 0.003). Similarly, in stage IIIC1, the experimental group exhibited better results in the 3-year OS rate, 3-year PFS rate, 3-year in-field PFS rate, and 3-year out-field PFS rate compared to the control group (*p* = 0.024; *p* = 0.012; *p* = 0.011; *p* = 0.027), respectively. However, there was no significant difference between the two groups in stages IIIC2 and IVA (Figs. 3, 4, 5 and 6).

### Toxicity and safety

The study found that both the experimental and control groups experienced adverse reactions, including radiation dermatitis (23.5% vs 17.3%; *p* = 0.264), radiation cystitis (18.5% vs 19.4%; *p* = 0.866), radiation enteritis (63.9% vs 54.1%; *p* = 0.144), and bone marrow suppression (90.75% vs 91.8%;* p* = 0.779). However, there was no significant difference in the incidence of these reactions between the two groups, as shown in Table 5.

**Table 5:** Toxicity and safety

	EG(*N* = 119),n(%)	CG(*N* = 98), n(%)	*p* -value
Radiation dermatitis			
Grade 0	91 (76.5%)	81 (82.7%)	0.264
GradeI-II	23 (19.3%)	15 (15.3%)	0.438
Grade III-IV	5 (4.2%)	2 (2.0%)	0.461
Radiation cystitis			
Grade 0	97 (81.5%)	79 (80.6%)	0.866
GradeI-II	22 (18.5%)	19 (19.4%)	0.866
Grade III-IV	0 (0%)	0 (0%)	
Radiation enteritis			
Grade 0	43 (36.1%)	45 (45.9%)	0.144
GradeI-II	57 (47.9%)	45 (45.9%)	0.771
Grade III-IV	19 (16%)	8 (8.2%)	0.083
Bone Marrow suppression			
Grade 0	11 (9.25%)	8 (8.2%)	0.779
GradeI-II	83 (69.75%)	73 (74.5%)	0.439
Grade III-IV	25 (21.0%)	17 (17.3%)	0.497

*EG* The experimental group, *CG* The control group

## Discussion

Cervical cancer is a prevalent gynecological malignancy in China that poses a significant threat to women's health [1]. For patients with stage IIB-IVA primary cervical cancer, radical chemoradiotherapy is the treatment of choice [2]. Still, the risk of recurrence after treatment is high, with a median recurrence time of 7–36 months, a recurrence rate between 8–26%, pelvic recurrence in 14–57% of patients and distant or multiple sites in 15–61% of patients [8]. For patients with stage IIB-IVA after radical radiotherapy, the 3-year PFS rate was 74.4% (95% CI, 68.0%-79.8%) [3], and the 5-year overall survival rate was approximately 70% [2]. A recent study found that patients with cervical cancer who underwent radical radiotherapy for stage IB1-IIIB had a local PFS rate of 70%-80% at two years [9]. Unfortunately, there is no standard treatment for recurrent cervical cancer and the prognosis is poor [10]. These studies suggest that while radical radiotherapy can help control cervical cancer, it is not always effective in preventing pelvic recurrence, which is a significant factor in treatment failure.

Numerous studies indicate that the presence of LNM and parametrial infiltration are significant factors that affect the prognosis of cervical cancer. In a retrospective analysis conducted by McComas et al., it was found that LNM negatively affected survival (IIIC1 Hazard Ratio [HR] = 2.0, *p* < 0.001, IIIC2 HR = 3.9, *p* < 0.001) [11].In a study conducted by Wang et al., 1433 patients with cervical cancer who underwent radical radiotherapy were reviewed. The study found that the 3-year disease-free survival (DFS) for patients with local LNM was 58.0%, while the DFS for patients without LNM was 81.8% [12]. The prognosis of cervical cancer patients is not solely determined by the presence or absence of lymph node metastases, but also by the number and size of the metastases [13, 14]. PET/CT is currently the most accurate test available for detecting metastatic lymph nodes with noperable people, however, it has limitations. In one of the most recent collaborative studies, the sensitivity, specificity, positive predictive value, and negative predictive value of PET/CT were 33.3%, 94.2%, 53.8%, and 87.5%, respectively, for the detection of microscopic lymph node metastases [15]. To reduce the risk of lymphatic metastases, increasing the dose of irradiation to the area of positive lymph node drainage may be necessary. The degree of parametrial infiltration also has significant prognostic significance. In their study, Wright et al. utilized data from the US National Cancer Database to analyze the 5-year overall survival rates of patients with stage IIIA (40.7%), IIIB (41.4%), IIIC1 (60.8%), and IIIC2 (37.5%). Their findings indicated that patients with stage IIIC1 had a significantly better prognosis compared to those with stage IIIA and IIB [16]. Grigsby et al. reported that the 5-year PFS rates were 52%, 63%, and 36% for stage IIIB, IIIC1, and IIIC2, respectively. Notably, patients with stage IIIC1 had a better prognosis than those with stage IIIB [17]. The findings indicate that the new staging system contradicts the previously held principle that higher stage indicates worse prognosis. The presence of a large localised tumour (stage IIIB) may have more clinical relevance to prognosis than lymph node metastases. These results provide theoretical support for using IMRT-SIB to parametrial areas and positive lymph node drainage.

According to the NCCN guidelines, the current radiotherapy regimen for cervical cancer combines ERBT and ICBT [18]. ICBT, in particular, is a crucial part of radical radiotherapy and used in conjunction with high-dose radiation at the end of external irradiation, greatly enhancing the local control rate and surviva lrate in advanced cervical cancer [19]. The intracavitary rear-mounted radiation source's fixed location can cause issues such as inadequate radiation to the tumor and excessive radiation to surrounding normal tissues, which can result in severe complications [20]. Improving the effectiveness of cervical cancer treatment while minimizing side effects is crucial in reducing recurrence rates. While some studies have attempted to increase overall radiation dose to improve local control rates in the pelvis, research has shown that this approach does not lead to further improvements in local control rates but increases the risk of late complications [21]. However, traditional supplemental dosing techniques are limited to two-dimensional imaging which can lead to unpredictable dose distribution to both the tumor and surrounding OARs [22].So is a method available that allows for a more rational target area dose distribution and simpler target area dose calculation? IMRT-SIB, derived from IMRT, is an irradiation method that simultaneously delivers split doses to different target areas within the same irradiation field. This method has demonstrated better outcomes in breast, rectal, and head and neck squamous cell carcinomas [23–25], and has also been reported to be effective in treating cervical cancer. The National Comprehensive Cancer Network (NCCN) has recently updated its guidelines to include sib to positive lymph nodes and parametrial areas as a new treatment option for cervical cancer. This provides patients with more options for managing their condition [18].

According to the NCCN guidelines, a dose of external radiation of around 40–45 Gy is needed to cover minimally diseased lymph nodes. For large unresected limited lymph node lesions, an additional dose of 10–20 Gy can be administered. However, it is important to take into account the dose of brachytherapy [18].In a study conducted by Yunzhi Dang et al., 74 patients with stage IIB-IVB cervical cancer underwent IMRT with simultaneous dose increments for pelvic lymph nodes, pelvic field doses of 45–50 Gy/25 f,positive lymph nodes of 62.5 Gy/25 f and intracavitary brachytherapy of 24 Gy/3 f to 42 Gy/6 f. The study found that the 3-year local control, distant metastasis-free survival, and overall survival rates were 91.7%, 75.7%, and 71.4%, respectively. Additionally, there were no significant differences in side effects observed [26]. Patients with locally advanced cervical cancer were treated with a combination of IMRT/VMAT (45 Gy/25f), weekly cisplatin chemotherapy, and pushes to LNM (60 Gy/25f), followed by ICRT(28 Gy/4f). The 3-year OS rate, local recurrence-free survival rate, regional recurrence-free survival rate, and distant recurrence-free survival rate were 69%, 91%, 79%, and 77% in 23 patients, not statistically significant when compared to patients without LNM [27]. In a prospective study by Beriwal et al., 36 patients with stage IB2-IVA cervical cancer were treated with IMRT and concurrent cisplatin chemotherapy. The metastatic lymph node area received a synchronous increase to 55-60 Gy, and high-dose-rate ICBT was implemented simultaneously. 34 patients achieved CR, 11 experienced recurrences, 2 had in-field recurrences, and 9 developed out-field recurrences [28]. Most studies suggest that simultaneous boost to positive lymph nodes can improve the control rate of cervical cancer. However, it has not been confirmed whether this treatment can improve the prognosis of cervical cancer.

According to the NCCN guidelines, in cases where large parametrial/pelvic sidewall tumors are not adequately covered by ICBT, parametrial boost of 5–10 Gy may be considered at the end of total pelvic radiotherapy, but only in selected cases [18]. According to some scholars, interstitial brachytherapy (IB) is an effective treatment for cervical cancer. It can increase the dose of the cancerous area while decreasing the dose of nearby OARs [29]. In a study evaluating the efficacy of IB for patients with bulky (≥ 4 cm) and high-risk, stage IIB-IVB advanced cervical cancer, the four-year rates of local control, pelvic control, DFS, and OS were 100%, 100%, 81.6%, and 87.8%, respectively [30]. Although IB is a viable medical procedure, it should be noted that it is invasive and carries a significant risk of complications, including but not limited to infection and haemorrhage. Marnitz et al.reported on the feasibility of using helical tomotherapy with the SIB technique to enhance radiation dose. Their study demonstrated a low rate of acute toxic reactions [31]. Building upon the success of helical tomotherapy with SIB, we believe that using IMRT-SIB is feasible.

In this study, a new target range of CTV-hr was defined to reduce the recurrence rate of patients by increasing the radiation dose to high recurrence sites without changing the total cervical radiation dose. In our study, implementing a CTV-hr and performing IMRT-SIB can lead to a significant improvement in the PFS and OS rates of patients with stage IIB-IVA cancer. we conducted a stratified analysis and found a significant increase in the 3-year OS rate, PFS rate, in-field PFS rate, and out-field PFS rate in patients with IIB-IIIC1 compared to the control group. However, no significant difference was observed in patients with stage IIIC2 and IVA. One possible explanation for this phenomenon is that patients in this stage may have already developed metastasis to the para-aortic lymph nodes or infiltration of the pelvic organs beyond the true pelvic area. This can ultimately result in distant metastases occurring in a shorter period of time, leading to a poor prognosis.

The study has a few limitations that should be noted. Firstly, it is a retrospective study. Secondly, the FIGO 2018 staging system has revised lymph node metastasis, but some of the data and references in this study are based on the previous 2009 FIGO staging system. Thirdly, since the patient did not undergo lymph node biopsy, it is necessary to verify whether the lymph nodes were indeed metastatic. Therefore, a prospective trial is needed to confirm these findings.

## Conclusion

Based on the above aspects, setting up CTV-hr with IMRT-SIB can improve the prognosis of cervical cancer patients without increasing radiation side effects, especially for stage IIB-IIIC1 patients. Therefore,it is an effective and safe method and should be recommended.

## Data Availability

The datasets generated and/or analysed during the current study are not publicly available due The experiment is still in progress but are available from the corresponding author on reasonable request.

## Abbreviations

IMRT: Intensity-modulated radiation therapy
IMRT-SIB: Simultaneous integrated boost intensity-modulated radiotherapy
SIB: Simultaneous integrated boost.
CTV-hr: High-risk clinical target volume
RT: Radiotherapy
EBRT: External beam radiation therapy
EQD2: The equivalent dose in 2 Gy/f
ICBT: Intracavitary brachytherapy
CT: Computed tomography
MRI: Magnetic resonance imaging
GTV: Gross tumor volume
CTV: Clinical target volume
PTV: Planning target volume
OS: Overall survival
ORR: Objective remission rate
PFS: Progression-free survival
RTOG: Radiation therapy oncology group
OARs: Organs at risk
LNM: Lymph node metastasis
PALN: Para-aortic lymph node
CTV-T_HR_: High-risk clinical target volume for tumors in ICBT
CTV-T_IR_: Intermediate-risk clinical target volume in ICBT
EFRT: Extended field radiotherapy
D90: The lowest dose received by 90% of the target volume
GTV-n: Metastatic lymph nodes
PET/CT: Positron emission tomography/computedtomography
PET/MR: Positron emission tomography/magnetic resonance
IGRT: Image Guided Radiotherapy
NCCN: The National Comprehensive Cancer Network
RECIST: Response Evaluation Criteria in Solid Tumors
CTCAE: Common Terminology Criteria for Adverse Events
VMAT: Volumetric modulated arc therapy
